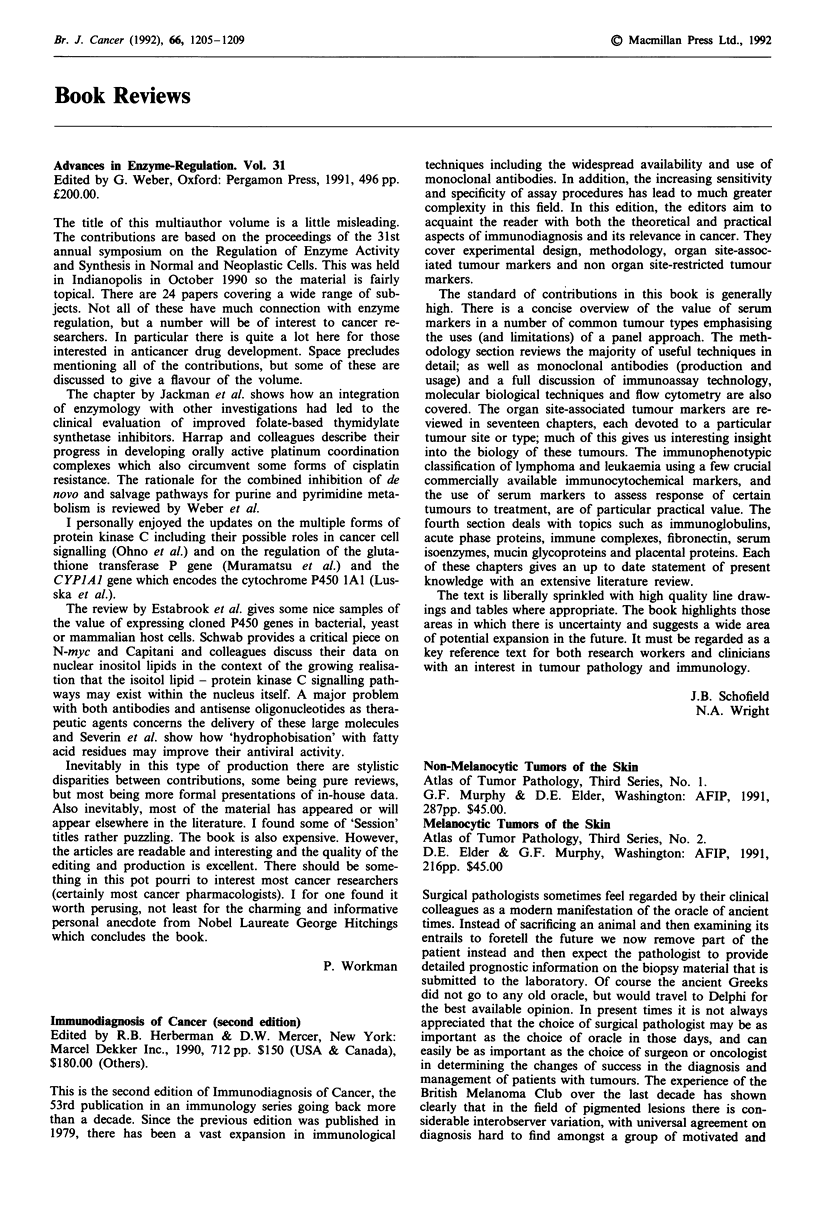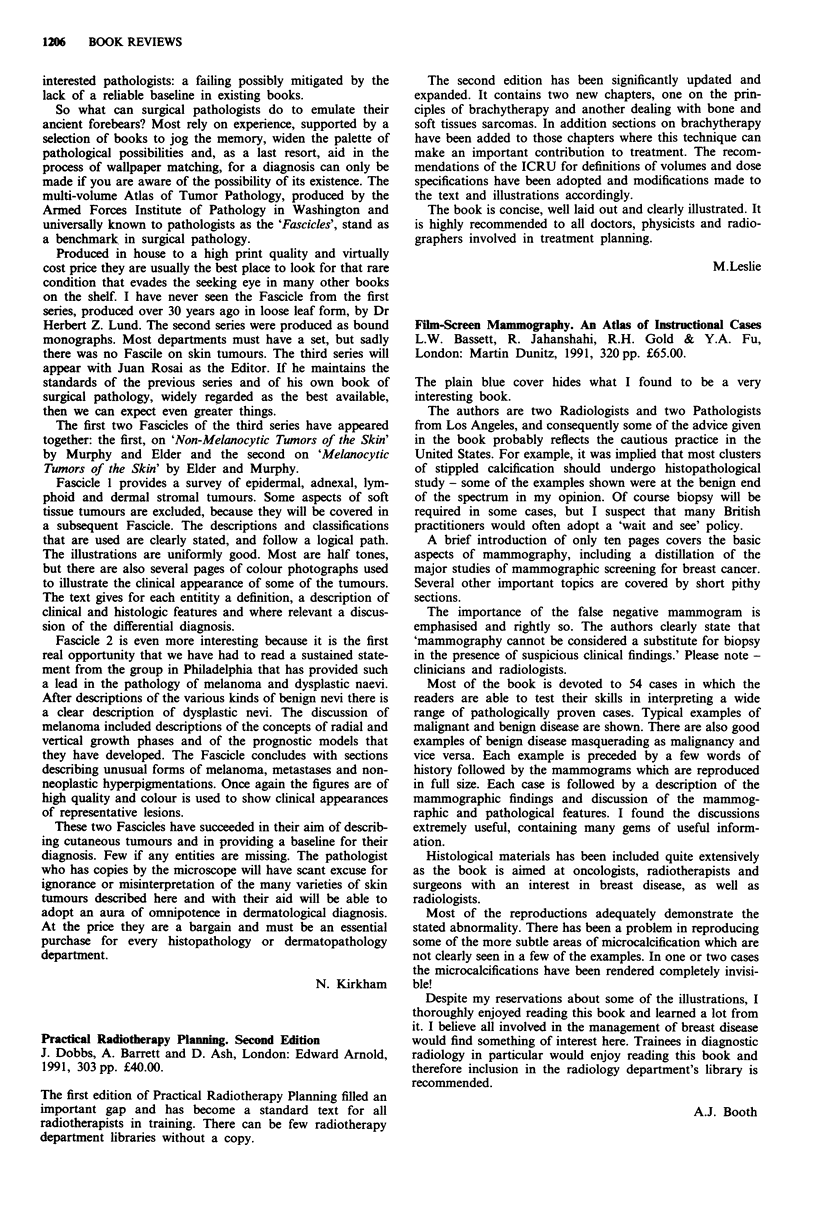# Non-Melanocytic Tumors of the Skin and Melanocytic Tumors of the Skin

**Published:** 1992-12

**Authors:** N. Kirkham


**Non-Melanocytic Tumors of the Skin**
Atlas of Tumor Pathology, Third Series, No. 1.
G.F. Murphy & D.E. Elder, Washington: AFIP, 1991, 287pp. $45.00.
**Melanocytic Tumors of the Skin**
Atlas of Tumor Pathology, Third Series, No. 2.
D.E. Elder & G.F. Murphy, Washington: AFIP, 1991, 216pp. $45.00

Surgical pathologists sometimes feel regarded by their clinical colleagues as a modern manifestation of the oracle of ancient times. Instead of sacrificing an animal and then examining its entrails to foretell the future we now remove part of the patient instead and then expect the pathologist to provide detailed prognostic information on the biopsy material that is submitted to the laboratory. Of course the ancient Greeks did not go to any old oracle, but would travel to Delphi for the best available opinion. In present times it is not always appreciated that the choice of surgical pathologist may be as important as the choice of oracle in those days, and can easily be as important as the choice of surgeon or oncologist in determining the changes of success in the diagnosis and management of patients with tumours. The experience of the British Melanoma Club over the last decade has shown clearly that in the field of pigmented lesions there is considerable interobserver variation, with universal agreement on diagnosis hard to find amongst a group of motivated and interested pathologists: a failing possibly mitigated by the lack of a reliable baseline in existing books.

So what can surgical pathologists do to emulate their ancient forebears? Most rely on experience, supported by a selection of books to jog the memory, widen the palette of pathological possibilities and, as a last resort, aid in the process of wallpaper matching, for a diagnosis can only be made if you are aware of the possibility of its existence. The multi-volume Atlas of Tumor Pathology, produced by the Armed Forces Institute of Pathology in Washington and universally known to pathologists as the '*Fascicles*', stand as a benchmark in surgical pathology.

Produced in house to a high print quality and virtually cost price they are usually the best place to look for that rare condition that evades the seeking eye in many other books on the shelf. I have never seen the Fascicle from the first series, produced over 30 years ago in loose leaf form, by Dr Herbert Z. Lund. The second series were produced as bound monographs. Most departments must have a set, but sadly there was no Fascile on skin tumours. The third series will appear with Juan Rosai as the Editor. If he maintains the standards of the previous series and of his own book of surgical pathology, widely regarded as the best available, then we can expect even greater things.

The first two Fascicles of the third series have appeared together: the first, on '*Non-Melanocytic Tumors of the Skin*' by Murphy and Elder and the second on '*Melanocytic Tumors of the Skin*' by Elder and Murphy.

Fascicle 1 provides a survey of epidermal, adnexal, lymphoid and dermal stromal tumours. Some aspects of soft tissue tumours are excluded, because they will be covered in a subsequent Fascicle. The descriptions and classifications that are used are clearly stated, and follow a logical path. The illustrations are uniformly good. Most are half tones, but there are also several pages of colour photographs used to illustrate the clinical appearance of some of the tumours. The text gives for each entity a definition, a description of clinical and histologic features and where relevant a discussion of the differential diagnosis.

Fascicle 2 is even more interesting because it is the first real opportunity that we have had to read a sustained statement from the group in Philadelphia that has provided such a lead in the pathology of melanoma and dysplastic naevi. After descriptions of the various kinds of benign nevi there is a clear description of dysplastic nevi. The discussion of melanoma included descriptions of the concepts of radial and vertical growth phases and of the prognostic models that they have developed. The Fascicle concludes with sections describing unusual forms of melanoma, metastases and non-neoplastic hyperpigmentations. Once again the figures are of high quality and colour is used to show clinical appearances of representative lesions.

These two Fascicles have succeeded in their aim of describing cutaneous tumours and in providing a baseline for their diagnosis. Few if any entities are missing. The pathologist who has copies by the microscope will have scant excuse for ignorance or misinterpretation of the many varieties of skin tumours described here and with their aid will be able to adopt an aura of omnipotence in dermatological diagnosis. At the price they are a bargain and must be an essential purchase for every histopathology or dermatopathology department.

N. Kirkham